# The online use of Violence and Journey metaphors by patients with cancer, as compared with health professionals: a mixed methods study

**DOI:** 10.1136/bmjspcare-2014-000785

**Published:** 2015-03-05

**Authors:** Elena Semino, Zsófia Demjén, Jane Demmen, Veronika Koller, Sheila Payne, Andrew Hardie, Paul Rayson

**Affiliations:** 1 Department of Linguistics and English Language, Lancaster University, Lancaster, UK; 2 Department of Applied Linguistics and English Language, The Open University, Milton Keynes, UK; 3 Department of Linguistics and Modern Languages, University of Huddersfield, Huddersfield, UK; 4 International Observatory on End of Life Care, Lancaster University, Lancaster, UK; 5 School of Computing and Communications, Lancaster University, Lancaster, UK

**Keywords:** Cancer, Communication

## Abstract

**Objective:**

To compare the frequencies with which patients with cancer and health professionals use Violence and Journey metaphors when writing online; and to investigate the use of these metaphors by patients with cancer, in view of critiques of war-related metaphors for cancer and the adoption of the notion of the ‘cancer journey’ in UK policy documents.

**Design:**

Computer-assisted quantitative and qualitative study of two data sets totalling 753 302 words.

**Setting:**

A UK-based online forum for patients with cancer (500 134 words) and a UK-based website for health professionals (253 168 words).

**Participants:**

56 patients with cancer writing online between 2007 and 2012; and 307 health professionals writing online between 2008 and 2013.

**Results:**

Patients with cancer use both Violence metaphors and Journey metaphors approximately 1.5 times per 1000 words to describe their illness experience. In similar online writing, health professionals use each type of metaphor significantly less frequently. Patients’ Violence metaphors can express and reinforce negative feelings, but they can also be used in empowering ways. Journey metaphors can express and reinforce positive feelings, but can also be used in disempowering ways.

**Conclusions:**

Violence metaphors are not by default negative and Journey metaphors are not by default a positive means of conceptualising cancer. A blanket rejection of Violence metaphors and an uncritical promotion of Journey metaphors would deprive patients of the positive functions of the former and ignore the potential pitfalls of the latter. Instead, greater awareness of the function (empowering or disempowering) of patients’ metaphor use can lead to more effective communication about the experience of cancer.

## Introduction

The shortcomings of war-related metaphors for cancer have been discussed in previous research and in the media.^1–3^ Recent UK policy documents, such as the 2007 NHS Cancer Reform Strategy, have avoided these metaphors in favour of the notion of cancer as a ‘journey’.^4^ Little is known, however, about how and to what extent patients use these different metaphors for their illness experiences.

This paper reports the results of a computer-assisted quantitative and qualitative study of the Violence and Journey metaphors used by patients with cancer in a 500 134-word ‘corpus’ (data set) of contributions to a UK-based online forum. We show how both types of metaphors are used significantly more frequently in this corpus than in a 253 168-word corpus of online writing by health professionals. We then focus on *how* patients use Violence and Journey metaphors, and show that both types can have empowering as well as disempowering functions.

A metaphor involves talking and potentially thinking about one thing in terms of another, on the basis of a perceived similarity between the two. A patient's description of herself as ‘fast becoming a chemo veteran’, for example, suggests the perception of a similarity between the experience of being treated with chemotherapy and the experience of fighting in a war.^5^

Metaphors have been found to occur between 3 and 18 times per 100 words.^6–8^ They are used to talk about abstract, complex, subjective and sensitive experiences in terms of more concrete, simpler, less subjective and less sensitive ones.^9^ Illness, death and the emotions around them are among the sensitive experiences that are often talked about metaphorically.^10^

Different metaphors ‘frame’ a topic in different ways, highlighting some aspects and backgrounding others.^9^ Expressions such as dying after a ‘long battle with cancer’ have become controversial precisely because of the framing that they may impose on the patient's experience: they have associations of violence and threat; they cast the patient in the aggressive role of a fighter; they suggest the presence of an enemy—the disease itself—inside the patient's body; and they associate not recovering with defeat.

The notion of cancer as a metaphorical journey is arguably the most prominent current alternative to Violence metaphors. The UK's 2007 Cancer Reform Strategy^4^ contains no instances of ‘battle’ or ‘war’, but includes repeated references to the patient's cancer ‘journey’, with different ‘pathways’ delineated as models of care. Journey metaphors frame the illness experience differently: they potentially cast the illness as a companion to live and travel with; and they do not involve the implication that not recovering amounts to personal failure.

While metaphors for cancer have been explored in a variety of contexts,^11–13^ patients’ spontaneous language use has not been studied systematically. We conducted a large-scale investigation of the Violence and Journey metaphors that patients with cancer use when writing online; we compared their frequencies with those we found in online writing by health professionals; and we considered their implications for empowering or disempowering the patients themselves. We show that Violence metaphors are not necessarily negative and Journey metaphors are not necessarily positive conceptualisations of cancer. The key issue, rather, is the function of a particular framing in a given context.

## Methods

### Design and setting

As part of the project ‘Metaphor in End-of-Life Care’,^14^ we studied the use of Violence and Journey metaphors in the following two data sets, or ‘corpora’:

- 500 134 words of online forum contributions by patients on a UK-based website dedicated to cancer;
- 253 168 words of online forum contributions, blog entries and comments by health professionals on a UK-based website.

All material was publicly accessible, but only registered members could contribute to the online fora. For our purposes, the spontaneity of expression allowed by anonymous online writing outweighs the lack of systematic demographic information for contributors.^15^ ^16^

### Sampling and data collection

Fifty-six contributors to the patient online forum were included in our corpus according to the following criteria:

- They described themselves as having received a terminal cancer diagnosis, or discussed palliative or end-of-life care; and
- They posted at least 50 contributions to the forum between 2007 and 2012.

To reduce the data sample to the desired size of approximately 500 000 words, we scaled the patients’ contributions down proportionally according to the total size of each user's contributions, taken from the point at which the patients began discussing end-of-life care (but with a minimum of 1000 words per patient).

For comparison purposes, 253 168 words were downloaded from a UK-based website for health professionals, consisting of online forum contributions, blog entries and comments about terminal illness, palliative care and end-of-life care. This data set spanned the period 2008–2013, was primarily concerned with cancer and included contributions from 307 health professionals, most of whom identified themselves as physicians.

The data were mass-downloaded using a bespoke computer application, and stored in a format appropriate for exploration by the software tools developed within Corpus Linguistics.^17^

### Analysis

A 15 000-word sample from each corpus was analysed manually in order to (1) identify metaphorical expressions according to a well-established procedure,^18^ and (2) allocate each expression to a ‘semantic field’ corresponding to its literal meaning (eg, ‘veteran’ in ‘a chemo veteran’ was identified as a metaphor and allocated to the semantic field ‘War’). This phase of the analysis was carried out by three team members: the main analyst's codings were independently verified by two other team members to ensure accuracy and consistency.

This sample analysis resulted in a list of linguistic expressions and semantic fields to be investigated in the two complete corpora. The online software Wmatrix was used for this purpose.^19^ ^20^ This tool allowed us to search for (A) all instances of words that we identified as potentially relevant metaphors in the sample analysis (eg, ‘weapon’); and (B) all instances of words that the in-built lexicon categorised under particular semantic fields (eg, the semantic field ‘Warfare’). The latter function is distinctive to our approach to large-scale metaphor analysis.^21^ The resulting lists of expressions were exported into spreadsheets and coded for metaphoricity by a team member. To ensure accuracy and consistency, three further team members independently verified and agreed on the codings in each spreadsheet.

In this study, we consider the metaphorical expressions in our two complete corpora that relate to the experience of cancer, and that have literal meanings that can be subsumed under the semantic fields of Violence and Journey. The search for potential Violence metaphors was conducted by searching for relevant semantic fields in the data (see (B) above). The search for potential Journey metaphors was conducted by means of a combination of word-level searches (see (A) above) and semantic-field searches (see (B) above), due to the high frequency of movement-related vocabulary in English (eg, ‘through’, ‘going to’).

We first calculated and compared the frequencies of Violence and Journey metaphors in the two data sets. We then investigated in detail the ways in which these metaphors are used by patients to express their experiences. In particular, we considered: (1) what aspects of the patients’ experience are described in terms of Violence or Journey; and (2) the framings provided by different uses of metaphor, particularly in terms of the patient's empowerment and disempowerment in the context of the illness, and the associated emotions. We define empowerment and disempowerment as an increase or decrease in the degree of *agency* that the patient has, or perceives him/herself to have, as manifest in the metaphors and their co-text. This involves the (perceived) ability to control or react to events for one's own benefit, where this ability is desired by the patient and not externally imposed.

## Results

### Quantitative findings

**Figure 1:**
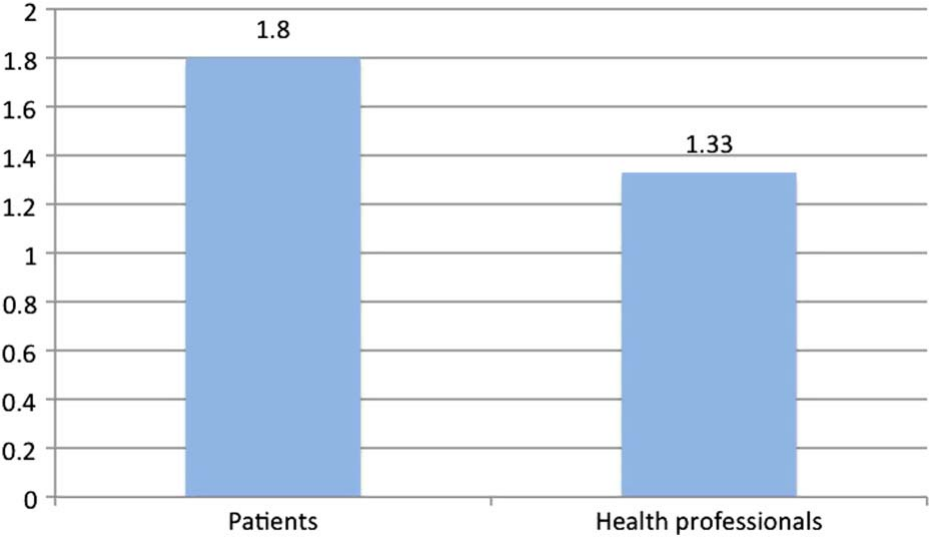
Frequencies per 1000 words of Violence metaphors in the Patient corpus and the Health Professional corpus.

Figure 1 provides normalised frequencies per 1000 words of occurrences of relevant Violence metaphors in the Patient corpus and the Health Professional corpus. The higher frequency in the Patient corpus is statistically significant at p<0.0001.

**Figure 2:**
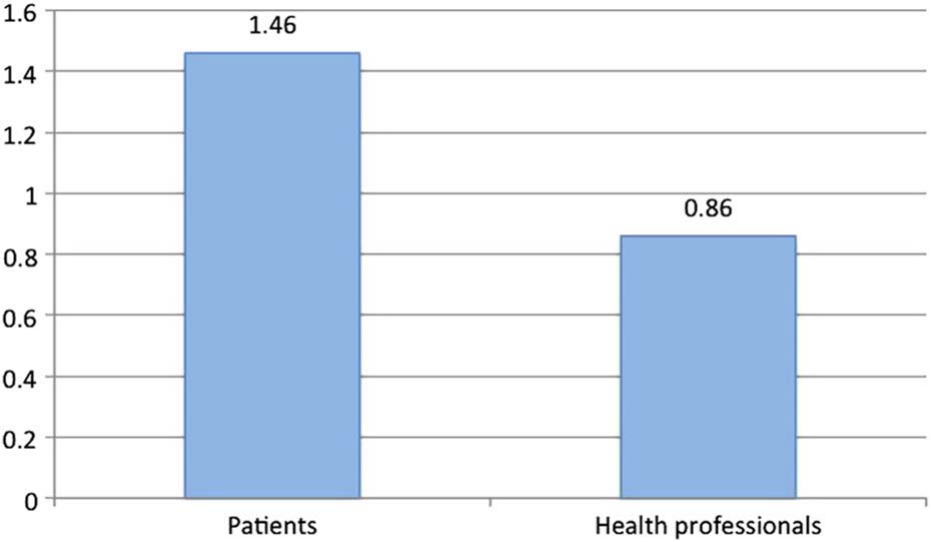
Frequencies per 1000 words of Journey metaphors in the Patient corpus and the Health Professional corpus.

Figure 2 provides normalised frequencies per 1000 words of occurrences of relevant Journey metaphors in the Patient corpus and the Health Professional corpus. The higher frequency in the Patient corpus is statistically significant at p<0.0001.

The fact that patients use both types of metaphors significantly more frequently than health professionals raises further questions. This is especially noteworthy considering that healthcare professionals use other types of metaphors as frequently as patients do (eg, Machine metaphors), or even more frequently (eg, Openness metaphors).

### Qualitative findings

As summarised in table 1, patients can use both Violence and Journey metaphors in empowering and disempowering ways.

**Table 1:** Summary of empowering and disempowering Violence and Journey metaphors

	Violence scenarios	Journey scenarios
Patient as disempowered	Disease fighting the patient Patient unsuccessfully fighting the disease Treatment fighting the patient	Patient as a traveller on a difficult journey Patient travelling without control over their journey
Patient as empowered	Patient successfully fighting the disease Patient successfully fighting health professionals Mutual encouragement and solidarity	Patient as a traveller in charge of the journey Patients as travelling companions

### Qualitative findings: Violence metaphors

Patients’ Violence metaphors can be used in both empowering and disempowering ways.

#### Violence metaphors and disempowerment

Patients’ Violence metaphors suggest several kinds of scenarios in which the patient appears in a disempowered position.

##### The disease fighting the patient

When Violence metaphors express patients’ perception of their illness, cancer can be described as ‘attacking from the inside’ and ‘invading’ the body. A patient describes her breast cancer as a ‘killer’ that ‘strangles and shocks your soul’. In such cases, the disease is presented as an aggressive opponent, while the patient is in a passive position. A particularly strong sense of vulnerability is expressed by patients who describe themselves as ‘time bombs’ while in remission.

##### The patient unsuccessfully fighting the disease

Violence metaphors are also used to express patients’ attempts to recover or to prolong their lives. When their condition does not improve, patients describe themselves as unsuccessful fighters. In some cases, this is because they are not prescribed the most effective treatment, as in: “it must be dispiriting when you are battling as hard as you can, not to be given the armour to fight in”. In other cases, lack of recovery is described in terms of defeat, as in: “I feel such a failure that I am not winning this battle”. This patient blames herself for the failure of her treatment, thus adding feelings of guilt to the emotional consequences of facing the terminal phase of the disease.

##### The treatment fighting the patient

Patients also use Violence metaphors to express their perception of the effects of cancer treatment. Chemotherapy is described as giving the patient's body ‘a hammering’ or ‘a battering’. One patient talks about her ‘normal little cells’ being ‘hit by a sledgehammer of both toxic chemicals and radiation’. These metaphors suggest a perception of vulnerability and passivity in relation to cancer treatment that is similar to what is expressed for the disease itself.

#### Violence metaphors and empowerment

Violence metaphors are also used by patients to express a perception of themselves as engaged and effective agents within the illness experience.

##### The patient successfully fighting the disease

Some patients describe themselves as ‘fighters’ in ways that suggest agency and pride, as in “I am such a fighter” and “my consultants recognised that I was a born fighter”. One patient attributes her ‘desire to fight and win’ to the fact that she is young and has a family. Another thanks other forum contributors for restoring her ‘fighting spirit’, so that she is ‘ready to kick some cancer butt’.

##### The patient successfully fighting health professionals

Some patients employ Violence metaphors to describe their interactions with health professionals. A patient describes a successful outcome in a consultation as ‘winning that battle’, while another uses the expression ‘twin attack’ to refer to how two family members managed to obtain a medical appointment. After expressing dissatisfaction with the stitching of her wound, another patient comments that now she has ‘another thing to beat my surgeon up about’.

##### Mutual encouragement and solidarity

Violence metaphors are sometimes used by forum contributors to encourage and motivate one another. Some patients praise others for being ‘fighters’ and for ‘winning the battle’ against cancer. Expressions such as ‘Soldier on everybody’ occur at the end of some posts. Contributors to one thread (including both women and men) jokingly use army titles such as ‘Captain’ for one another; one particular patient says that she would ‘promote’ another if he had not already ‘reached top rank’.

Although this part of our study was qualitative in nature, 42 out of 100 randomly selected Violence metaphors were found to be used in a potentially empowering way, and 38 in a potentially disempowering way (the remaining 20 instances did not clearly fall under either category).

### Qualitative findings: Journey metaphors

Like Violence metaphors, Journey metaphors are used by patients in our data in both empowering and disempowering ways.

#### Journey metaphors and empowerment

Journey metaphors work in potentially empowering ways when they are used to convey a sense of purpose, control and companionship.

##### The patient as a traveller in charge of the journey

Some patients use Journey metaphors to express a sense of control over their cancer experience, and occasionally to point out some positive aspects of being ill, as in: “My journey may not be smooth but it certainly makes me look up and take notice of the scenery!” Another patient points out that, even when he thinks he has “gone as far as” he can, he has a “happy surprise by being able to push myself that little bit extra”.

##### Patients as travelling companions

Patients often use Journey metaphors to express a sense of group solidarity among forum users, and to encourage one another. This involves Journey scenarios in which patients travel together, or where patients with earlier diagnoses ‘guide’ those with more recent diagnoses. One patient comments that “rocks in our paths are easier to handle when we're all in it together”, and that “the best people to help you are the ones who've been there before or are heading there with you”. Journey metaphors are also used as mutually encouraging greetings, as in ‘Safe journey’.

### Journey metaphors and disempowerment

Journey metaphors are also used by patients in our data to express a sense of lack of acceptance of or control over their situation.

#### The patient as a traveller on a difficult journey

Some patients use Journey metaphors to emphasise the overwhelming difficulties they face as cancer sufferers. One patient comments that the journey has “many twists and turns that means that no two people go exactly the same route”, and adds that having cancer “is like trying to drive a coach and horses uphill with no back wheels on the coach”. Another comments that she has ‘not done so well’ on her ‘‘cancer journey’ through the wilderness’ of her local hospital.

#### The patient travelling without control over their journey

Many patients express the problems they have accepting their situation by describing themselves as travelling against their will. One patient talks about a ‘reluctant journey’, while another wonders how she can ‘navigate this road’ that she does ‘not even want to be on’. Some patients describe themselves as being on a journey that they cannot control. One forum user talks about other patients as “passengers” who are ‘nearing the end of their journey’ or have ‘finished their journey’.

In a sample of 100 randomly selected Journey metaphors, 26 were used in a potentially empowering way, while 39 suggested that the patient was in a disempowered position (the remaining 35 instances did not clearly fall under either category).

## Discussion

To the best of our knowledge, this is the first large-scale study of the use of Violence and Journey metaphors by patients with cancer, and the first systematic comparison of differences in frequency of use between patients and health professionals.

The higher frequency of both types of metaphor in the patient data suggests that they are helpful in expressing different aspects of the patients’ experience. The fact that health professionals use Violence metaphors less frequently may reflect an awareness of their potential shortcomings, and, more generally, of the importance of using appropriate and sensitive metaphors in relation to illness. As for function, our study confirms that Violence and Journey metaphors facilitate different ways of framing the patients’ experiences. Violence metaphors present the experience as an antagonistic one, in which the patient faces an opponent, whether this be the illness, the treatment, health professionals, etc. Violence metaphors may therefore both reflect and reinforce an adversarial approach to the cancer experience, as well as feelings of vulnerability, passivity, impending threat and, most negatively, personal failure if the disease is found to be incurable. In contrast, Journey metaphors can present the experience of illness as a process that is shared by others with similar diagnoses, or with family and friends. The patient can therefore take on a role that is active without being oppositional, while setbacks are not necessarily as catastrophic and irreversible as military defeats or bomb explosions.

However, our data also show that the specific framings provided by both Violence and Journey metaphors vary depending on who uses them and how. This variation relates particularly to: the degree of agency that the patient feels they have to act in their own interest; the extent to which this degree of agency is welcome; and the associated emotions. Some uses of Violence metaphors suggest that some patients find meaning, purpose and a sense of pride and identity in approaching the illness experience as a fight. Conversely, Journey metaphors can be used to convey feelings of passivity, lack of acceptance and pessimism.

Overall, therefore, Violence metaphors are not always negative, while Journey metaphors are not always positive. Metaphor use should be evaluated on the basis of its empowering or disempowering function, and associated emotions, in particular contexts.

### Strengths and weaknesses of the study

The use of online forum data allowed us to investigate the language that patients use in an informal and supportive environment, in which they are able to control how much information they disclose about themselves. By combining manual and computer-aided analysis, we identified a larger number of Violence and Journey metaphors than is possible by more traditional approaches.

However, our data only represent people who are comfortable with writing about their experiences online. Moreover, the lack of demographic information for forum contributors limits our ability to generalise our findings. Finally, our suggestions about the extent to which different uses of metaphor are empowering or disempowering are based on textual analysis alone, and cannot be confirmed by interviewing the patients or by considering the stage and seriousness of their condition. Our reliance on patients’ own self-descriptions also means that we cannot verify their diagnoses or disease status.

### Meaning of the study: implications for health professionals and policymakers

Our study provides enough evidence of the disadvantages and potential disempowering effects of Violence metaphors to support their exclusion from policy documents, and to discourage physicians from introducing them in interactions with patients. It can be particularly harmful for patients to have the role of ‘fighter’ imposed on them by external pressures, whether from relatives, health professionals, charity campaigns or a more general sense that refusing to ‘fight’ suggests a lack of determination and moral fibre. Our study also confirms that Journey metaphors can be employed to suggest a positive, empowering approach to the cancer experience, in which the patient feels a sense of companionship with others and can choose the degree of control he or she wishes to have in the decisions and processes that affect them. In this sense, the adoption of the metaphor of a ‘cancer journey’ can be appropriate and effective.

However, our study also shows that Violence metaphors are not by default negative and Journey metaphors are not by default a positive means of talking and thinking about cancer. Patients frequently use Violence metaphors in ways that seem to empower and motivate them, while their use of Journey metaphors can sometimes indicate a sense of disempowerment. Furthermore, patients also use Violence metaphors to describe their perception of difficulties and problems in the healthcare system, which may need to be addressed in the provision of healthcare. Hence, a blanket rejection of Violence metaphors would deprive some patients of the positive functions that these metaphors can have, while an uncritical promotion of Journey metaphors overlooks the negative ways in which they can be used.

We argue therefore that metaphor use should not be assessed only on the basis of type (Violence or Journey), but on the basis of its function (empowering or disempowering, and emotional associations). This has implications for training and practice in healthcare communication. By developing the skills of noticing and attending to patients’ metaphors, health professionals can be in a better position to question metaphors that seem to have negative, disempowering effects, and to accept or even adopt metaphors that seem to work in positive, empowering ways.

### Unanswered questions and future research

Further research is needed to identify variation in metaphor use and appropriateness for different groups of patients, depending on age, gender, cultural background, illness, stage of disease and so on. This would require the use of a data collection method that includes detailed demographic information. Future work is also needed to gather patients’ own perceptions of the metaphors they use and are exposed to during their illness experience.

In conclusion, patients with cancer use both Violence and Journey metaphors, and do so more frequently than health professionals. Both Violence and Journey metaphors can suggest empowerment as well as disempowerment, depending on who uses them and how. It follows that communication in cancer care could benefit from a focus on the function (empowering or disempowering) of individual metaphor uses rather than on their type (Violence or Journey).
